# Machine Learning Models Using General and Tissue-Specific Feature Extractors for Accurate Subtyping of Biopsy Samples: Advancing Lung Cancer Diagnosis in Latin America

**DOI:** 10.1016/j.jtocrr.2025.100906

**Published:** 2025-09-18

**Authors:** Viviane Teixeira Loiola de Alencar, Felipe Navarro Balbino Alves, Guilherme de Souza Velozo, Luiz Edmundo Lopes Mizutani, Iusta Caminha, Gabriel Barbosa Silva, Vladmir Cláudio Cordeiro de Lima, Fábio Rocha Fernandes Távora

**Affiliations:** a Research and Development Department, Oncodata, São José dos Campos, Brazil; b Department of Medical Oncology, A.C. Camargo Cancer Center, São Paulo, Brazil; c Digital Pathology Department, Argos Anatomia Patológica, Fortaleza, Brazil; d Pathology Department, Federal University of Ceará, Fortaleza, Brazil

**Keywords:** Lung cancer, Digital pathology, Artificial intelligence, Machine learning, Deep learning, Histologic subtyping

## Abstract

**Introduction:**

Lung cancer is the leading cause of cancer-related deaths worldwide, with accurate histologic subtype classification critical for diagnosis and treatment planning. Diagnostic variability and resource disparities, particularly in underrepresented regions such as Latin America, pose substantial challenges. This study developed and evaluated novel artificial intelligence models trained on both global and Latin American pathology samples for subtype classification of hematoxylin and eosin (HE)–stained whole-slide images (WSIs).

**Methods:**

Two DinoV2-based feature extractors, LungDino and OncoDino, trained on large data sets for task-specific and general pathology applications, were developed. The training data set consisted of 1308 HE-stained WSIs, including 412 adenocarcinomas, 323 squamous cell carcinomas, 41 small cell carcinomas, and 532 benign tissue samples, sourced from The Cancer Genome Atlas and an in-house Latin American data set. A ResNet model trained on ImageNet served as the baseline. Models were evaluated on 79 Latin American WSIs using receiver operating characteristic curves, and heatmaps were generated for tumor localization.

**Results:**

The DinoV2-based models outperformed the ResNet baseline. LungDino achieved the highest overall performance, with area under the curves of 0.97 for adenocarcinoma and 0.96 for squamous cell carcinoma. OncoDino excelled in underrepresented categories, achieving an area under the curve of 0.99 for small cell carcinoma, demonstrating its generalizability. Both models generated interpretable heatmaps, with LungDino demonstrating precise tumor localization. In the subset of samples classified as poorly differentiated or undifferentiated in HE pathology reports, the DinoV2 models also maintained high classification performance.

**Conclusion:**

These findings underscore the effectiveness of task-specific and general feature extractors in delivering accurate, explainable results and address a gap in artificial intelligence–driven histopathology advancements, paving the way for future clinical applications.

## Introduction

Lung cancer is the most frequently diagnosed cancer globally, with nearly 2.5 million new cases annually, and it remains the leading cause of cancer-related mortality, accounting for approximately 1.8 million deaths each year.^1^ In Latin America, it ranks as the fourth most common cancer type, but it continues to be the leading cause of cancer-related deaths in the region.^1^ Although screening strategies have improved early-stage detection, nearly 70% of cases are still diagnosed at advanced stages, underscoring persistent challenges in early detection and diagnosis.^2^^,^^3^

Pathology is critical for the accurate diagnosis and treatment planning of patients with lung cancer. However, this process often depends on small biopsy samples, which may add complexity to diagnosis.^4^ Interpathologist variability in subtype classification ranges from 13% to 50%, with 29% to 85% of cases requiring additional immunohistochemical (IHC) staining to confirm a diagnosis, besides those initially performed for thyroid transcription factor—1 (TTF-1) and p40.^5^ These challenges are further compounded by the need to preserve tissue for further molecular analyses, particularly in nonsquamous NSCLC cases, which harbor most actionable driver-gene mutations.^6^^,^^7^

The global histopathology workforce faces mounting pressures, including a declining number of pathologists, an aging workforce, and increasing workload complexity. Evidence suggests that these issues are negatively affecting service delivery, patient care, and the well-being of pathologists.^8^ The shortage is particularly acute in Latin America, where there are only 17 pathologists per million people, compared with 50 to 65 per million in North America.^9^ Such disparities lead to delays in cancer diagnosis, with pathology results in some parts of Brazil, the region’s largest country, taking up to 20 business days to be delivered.^10^

Artificial intelligence (AI) offers promising tools to address these challenges by enhancing diagnostic accuracy and efficiency in pathology. Despite its potential, widespread implementation remains limited due to factors such as insufficient annotated data for training and validation, the opaque nature of black-box models, and biases in data sets which often lack representation from under-resourced regions. Notably, clinical AI data sets are disproportionately dominated by data from the United States and China, which may restrict their relevance to other populations.^11^^,^^12^ Furthermore, the cost of implementation, including routine digitization and access to AI algorithms, can be prohibitive, particularly in low-resource settings, underscoring the need for cost-effective and scalable solutions.

This study aimed to evaluate the effectiveness of a machine learning tool trained on both regional and global data sets in accurately classifying hematoxylin and eosin (HE)–stained lung biopsy samples from a real-world Latin American data set into the following four categories: adenocarcinoma, squamous cell carcinoma, small cell carcinoma, and benign tissue.

## Materials and Methods

### Data Collection and Preprocessing for the Classification Model

The training data set for the lung subtype classification model consisted of adenocarcinoma, squamous cell carcinoma, small cell carcinoma, and benign tissue samples obtained from The Cancer Genome Atlas (TCGA)^13^ and an in-house data set, which was developed using fully anonymized patient data to ensure confidentiality. TCGA samples were obtained from surgical resections, whereas the in-house data set comprised biopsy specimens. To address the imbalance caused by the smaller number of small cell carcinoma cases, oversampling techniques were applied to the minority classes, ensuring balanced representation during model training. This study was conducted in accordance with ethical guidelines and approved by the local Institutional Ethics Committee (CAAE: 69402823.6.0000.504). All patient data used in this research were anonymized, and the analysis did not include any identifiable information. Given the retrospective nature of the study and the use of fully deidentified data, the requirement for individual informed consent was waived by the ethics committee.

### Feature Extraction

Two different feature extractors were developed and three were tested. The first was a ResNet model^14^ trained on the ImageNet data set,^15^ which served as a baseline. ImageNet is a large-scale, hierarchically structured image database built on the WordNet ontology. It contains millions of high-resolution, manually annotated images organized across thousands of synsets, each representing a distinct semantic concept. Designed to support research in computer vision, ImageNet has played a critical role in advancing object recognition and image classification tasks due to its scale, diversity, and semantic richness.^15^

The second and third feature extractors, developed in this study, were based on DinoV2,^16^ a self-supervised learning framework designed to train vision transformers (ViTs) without requiring labeled data, making it particularly suitable for medical imaging tasks where annotations are often limited. Whole-slide images (WSIs) used for training were divided into fix-sized patches of 0.3168 mm × 0.3168 mm, which were redimensioned to 224 × 224 pixels to enable efficient processing by the DinoV2 architecture. Rather than using a pure sliding window or manual annotation, a tissue detection strategy based on color filtering was used to identify regions of interest (ROIs) containing viable tissue. Patches were then sampled automatically from these ROIs, allowing to focus on informative areas while avoiding background or empty regions.

Each DinoV2-based feature extractor was developed for a specific purpose:

• LungDino: Trained exclusively on 3210 lung tissue samples from TCGA. This model used a 1.2-terabyte data set consisting of 1,935,106 image patches extracted from 3215 HE-stained lung images.
• OncoDino: Trained on a more diverse data set, including patches from multiple tissue sites, to enhance generalizability across histologic patterns. This model used a 6.5-terabyte data set comprising 10,212,976 patches from 21,479 histology images, retrieved from TCGA^13^ (11,257), GTEx^17^ (6664), and an in-house data set (3558).

### Classification Models Development and Training

#### Data Sets for Training, Validation, and Testing of the Classification Models

The full data set for the classification model included cases from both TCGA and the in-house collection and was divided into the following three mutually exclusive subsets: training, validation, and testing. Data were randomly assigned to each subset, with strict patient-level separation to ensure that images from the same individual were not distributed across multiple subsets.

The training set was used to learn the model parameters, whereas the validation set supported hyperparameter tuning and early stopping to prevent overfitting. The testing set, used exclusively for final performance evaluation, consisted entirely of real-world specimens from Latin America and was not used at any stage during model training or validation.

Among the testing samples, 54.4% were classified in the HE reports as poorly differentiated or undifferentiated neoplasms, 29.1% as negative for malignancy, and only 16.5% as clearly diagnosed adenocarcinoma or squamous cell carcinoma based on HE alone. Final characterization of these samples as adenocarcinoma, squamous cell carcinoma, or small cell carcinoma was determined by IHC.

Tables 1 and 2 summarize the distribution of images by data set subset and data source.

**Table 1:** Distribution of Specimens Across Training, Validation, and Testing Sets, Grouped by Data Source

Data Set	TCGA	In-House Data Set
Training	1105	210
Validation	538	37
Test	0	79

**Table 2:** Distribution of Specimens Across Training, Validation, and Testing Sets, Categorized by Data Source and Tissue Type

Data Set by Histology	TCGA	In-House Data Set	Total
Adenocarcinoma			
Training	470	69	539
Validation	186	9	195
Test	0	26	26
Squamous cell carcinoma			
Training	370	42	412
Validation	221	7	228
Test	0	16	16
Small cell carcinoma			
Training	0	41	41
Validation	0	7	7
Test	0	14	14
Benign tissue			
Training	265	58	323
Validation	131	14	145
Test	0	23	23

#### Development of the Classification Model for Lung Tissue

The classification model was trained on a data set of 1315 HE-stained lung tissue samples, sourced from TCGA (n = 1105)^13^ and an in-house data set (n = 210), which represented a smaller yet critical portion of the training data set. The WSIs were also divided into patches, as described in the Feature Extraction section, and no manual selection was performed by pathologists. The model was trained using a weakly supervised multiple instance learning (MIL) approach, in which each patch inherited the slide-level label. Feature representations of the patches were aggregated using a modified version of the CLAM^18^ (Cluster-Consistent Attention-based Multiple Instance Learning) model, optimizing its attention mechanisms and cost functions for improved performance.

These samples were categorized as follows:

• TCGA: 470 adenocarcinomas, 370 squamous cell carcinomas, zero small cell carcinomas, and 265 benign tissue samples.
• In-house data set: 69 adenocarcinomas, 42 squamous cell carcinomas, 41 small cell carcinomas, and 58 benign tissue samples.

To address the imbalance caused by the limited representation of small cell carcinoma and the relatively smaller size of the in-house data set, oversampling techniques were applied to ensure adequate representation of minority classes and improve model performance.

The validation set comprised 575 images drawn from both TCGA and the in-house data set, as outlined in Tables 1 and 2. During training, the mean area under the curve (AUC) across all classes was calculated on the validation set at the end of each epoch. The model checkpoint with the highest mean AUC was selected. An early stopping strategy was applied: if no improvement in the mean AUC was observed after 10 consecutive epochs, training was halted.

#### Testing of the Models' Performance

**Figure 1:**
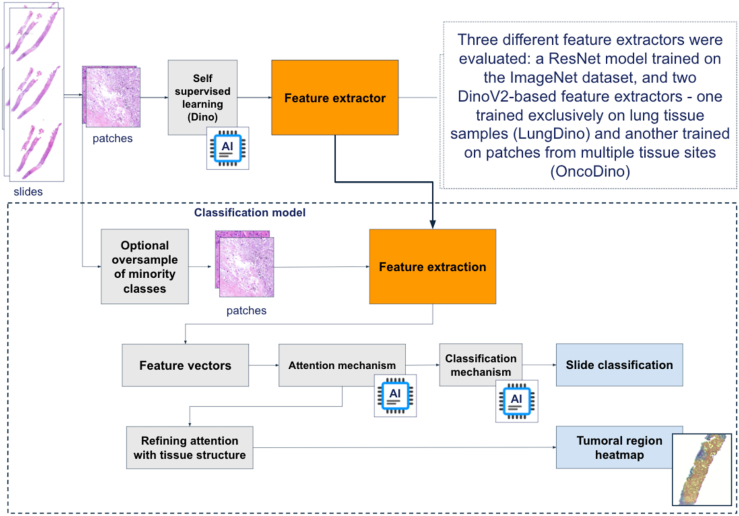
Workflow for feature extraction and classification. Overview of the pipeline used for training and testing. WSIs were divided into patches and processed through self-supervised learning using DinoV2 to generate feature extractors. Three feature extractors were tested: ResNet (trained on ImageNet), LungDino (trained on lung tissue samples), and OncoDino (trained on patches from multiple tissue sites). The classification model incorporated optional oversampling for minority classes, extracted feature vectors, and applied attention and classification mechanisms to generate final slide classifications and tumoral region heatmaps. WSI, whole-slide image.

The performance of LungDino and OncoDino feature extractors was compared against ResNet50 to evaluate the added value of task-specific and general feature representations for the classification model. Figure 1 illustrates the workflow for feature extraction and classification.

The test data set comprised 79 biopsy samples from a real-world Latin American data set, not used for training or validation. Diagnostic accuracy for these samples was independently validated using previously performed IHC tests. Model performance was assessed using the AUC metric, based on the ability to classify biopsy samples into the following four categories: adenocarcinoma, squamous cell carcinoma, small cell carcinoma, and benign tissue.

### Heatmap Generation

The most promising models were further used to generate heatmaps on WSIs, identifying tissue regions most relevant to the final classifications. These heatmaps highlight probable tumor areas, providing an explainable visualization of the model's decision-making process. Heatmap intensity ranges from dark blue (0%) to bright yellow (100%), corresponding to the attention or relevance score attributed by the model to each region. This feature is intended to enhance interpretability for pathologists, facilitating potential future applications in clinical practice. For this analysis, two random WSIs were selected, and an expert thoracic pathologist annotated tumor areas on these images to serve as ground truth for comparison. The annotation was performed in a blinded manner, ensuring that the pathologist did not have prior access to the models’ results. To improve the visibility of histologic features, figures displaying the pathology slides were presented at higher magnification, focusing on regions of interest.

## Results

### Classification Performance Across Models

**Figure 2:**
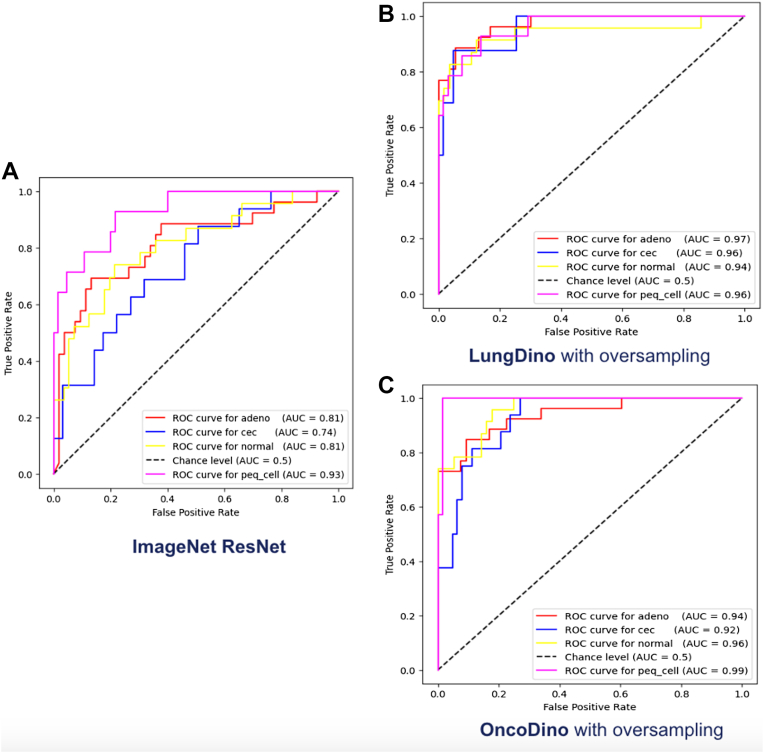
ROC curves for ResNet and feature extractors with oversampling. ROC curves demonstrating the classification performance of the models for four classes using different feature extractors on the test data set—adenocarcinoma (adeno), squamous cell carcinoma (cec), small cell carcinoma (peq_cell), and benign tissue (normal). (*A*) ResNet trained on ImageNet. (*B*) LungDino with oversampling. (*C*) OncoDino with oversampling. ROC, receiver operating characteristic.

**Figure 3:**
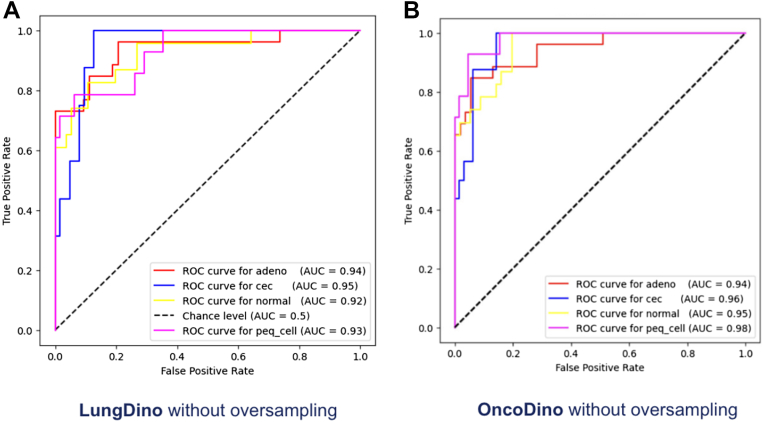
ROC curves for feature extractors without oversampling. ROC curves demonstrating the classification performance of the two DinoV2-based feature extractors without oversampling on the test data set, considering four classes—adenocarcinoma (adeno), squamous cell carcinoma (cec), small cell carcinoma (peq_cell), and benign tissue (normal). (*A*) LungDino without oversampling. (*B*) OncoDino without oversampling. ROC, receiver operating characteristic.

The performance of the classification models was evaluated using different feature extractors—ResNet trained on ImageNet, LungDino (with and without oversampling), and OncoDino (with and without oversampling)—on a test data set of 79 real-world Latin American lung biopsy samples. The models' ability to classify samples into four categories—adenocarcinoma (adeno), squamous cell carcinoma (cec), benign tissue (normal), and small cell carcinoma (peq_cell)—was assessed using receiver operating characteristic (ROC) curves and the AUC values, as found in Figures 2 and 3.

#### ImageNet ResNet

The ResNet baseline achieved moderate performance, with AUC values of 0.81 for adenocarcinoma, 0.74 for squamous cell carcinoma, 0.81 for benign tissue, and 0.93 for small cell carcinoma (Fig. 2*A*).

#### LungDino

With Oversampling: The inclusion of oversampling to address class imbalance further enhanced LungDino’s performance. AUC values reached 0.97 for adenocarcinoma, 0.96 for squamous cell carcinoma, 0.94 for benign tissue, and 0.96 for small cell carcinoma (Fig. 2*B*).

Without Oversampling: LungDino, trained on lung tissue samples from TCGA without oversampling, demonstrated significant improvement over the ResNet baseline. AUC values increased to 0.94 for adenocarcinoma, 0.95 for squamous cell carcinoma, 0.92 for benign tissue, and 0.93 for small cell carcinoma (Fig. 3*A*).

#### OncoDino

With Oversampling: OncoDino with oversampling demonstrated the best overall performance, achieving AUC values of 0.94 for adenocarcinoma, 0.92 for squamous cell carcinoma, 0.96 for benign tissue, and 0.99 for small cell carcinoma (Fig. 2*C*).

Without Oversampling: OncoDino, trained on a diverse data set including patches from multiple tissue types without oversampling, also achieved high AUC values at 0.94 for adenocarcinoma, 0.96 for squamous cell carcinoma, 0.95 for benign tissue, and 0.98 for small cell carcinoma (Fig. 3*B*).

#### Models’ Performance in Poorly Differentiated or Undifferentiated Neoplasms

**Figure 4:**
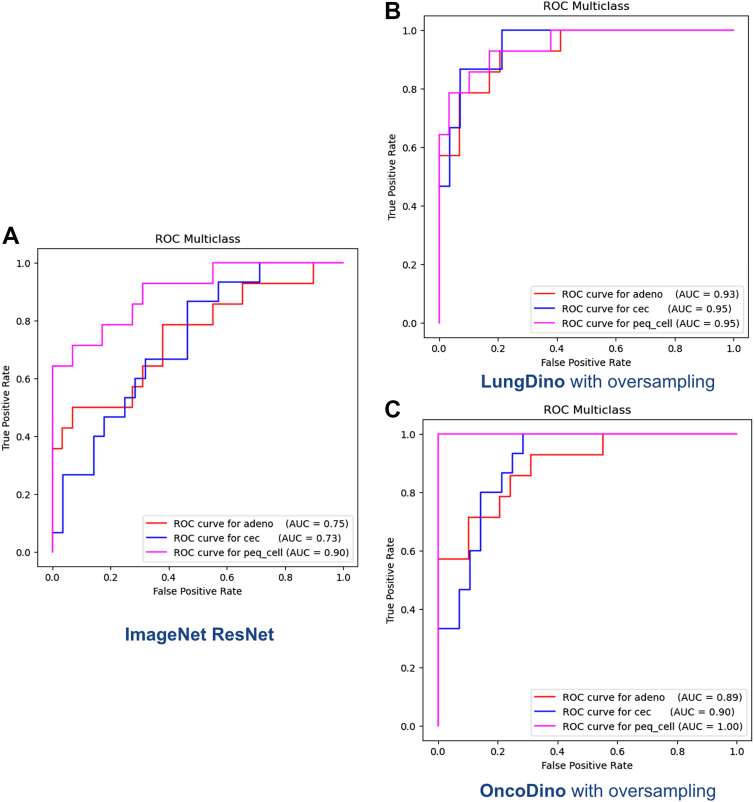
ROC curves for model performance on poorly differentiated or undifferentiated lung cancer cases. ROC curves evaluating the ability of each model to classify challenging neoplasms, originally diagnosed as poorly differentiated or undifferentiated on HE and reclassified using IHC as the reference standard, into adenocarcinoma (adeno), squamous cell carcinoma (cec), and small cell carcinoma (peq_cell). (*A*) ImageNet ResNet baseline. (*B*) LungDino with oversampling, trained on lung-specific data. (*C*) OncoDino with oversampling, trained on diverse tumor types. HE, hematoxylin and eosin; IHC, immunohistochemistry; ROC, receiver operating characteristic.

Given that poorly differentiated or undifferentiated neoplasms often represent the most diagnostically challenging cases in clinical practice, model performance was further evaluated on this subset within the test data set, reclassified using IHC as the reference standard. For this analysis, OncoDino and LungDino (with oversampling) and ResNet were assessed. The AUCs for classifying these challenging cases into adenocarcinoma, squamous cell carcinoma, or small cell carcinoma are as follows:

ResNet: adenocarcinoma 0.75, squamous cell carcinoma 0.73, small cell carcinoma 0.90 (Fig. 4*A*).

LungDino: adenocarcinoma 0.93, squamous cell carcinoma 0.95, small cell carcinoma 0.95 (Fig. 4*B*).

OncoDino: adenocarcinoma 0.89, squamous cell carcinoma 0.90, small cell carcinoma 1.00 (Fig. 4*C*).

#### Misclassified Samples

Among the misclassified slides, 78.6% were labeled in the HE reports as poorly differentiated NSCLC or undifferentiated neoplasm. Based on immunohistochemistry, 35.7% of these errors were ultimately diagnosed as squamous cell carcinoma. The remaining misclassified cases were distributed across primary lung adenocarcinoma, small cell carcinoma with neuroendocrine differentiation, and benign cases, each comprising approximately 21.4%. However, this study was not able to assess whether specific adenocarcinoma subtypes contributed disproportionately to misclassification, as subtyping was not possible in most misclassified cases.

### Heatmap Generation

**Figure 5:**
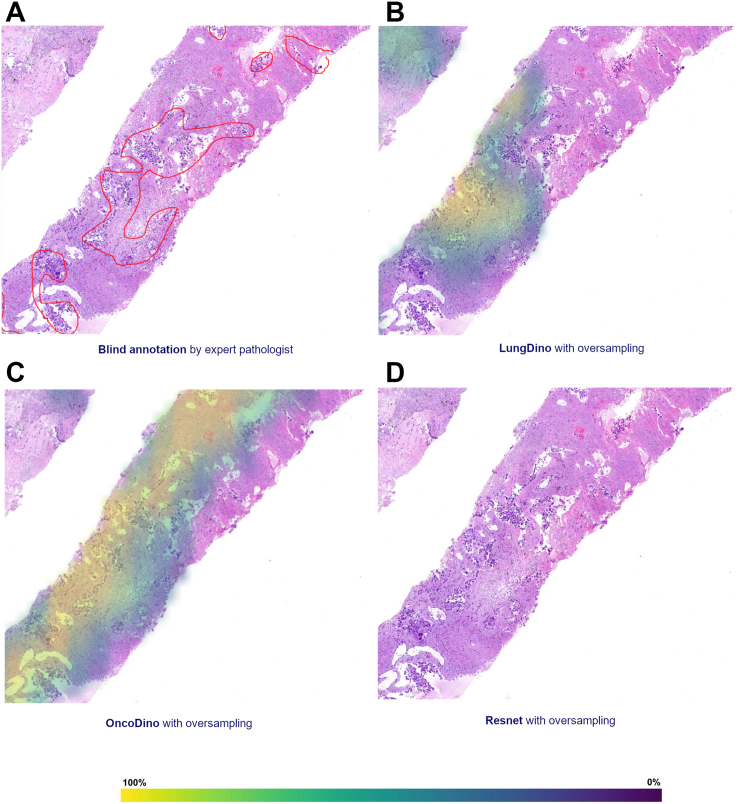
Tumor localization heatmaps generated by deep learning models in biopsy samples in comparison to expert pathologist annotation. Heatmaps indicate tissue areas most relevant for the final classification, highlighting probable tumor areas. The color gradient ranges from dark blue (0%) to bright yellow (100%), which corresponds to the attention or relevance score assigned by the model to each region. (*A*) Manual tumor annotation performed by a board-certified pathologist blinded to models’ outputs. (*B*) LungDino with oversampling. (*C*) OncoDino with oversampling. (*D*) The ResNet-based model, which did not produce a heatmap in this case.

**Figure 6:**
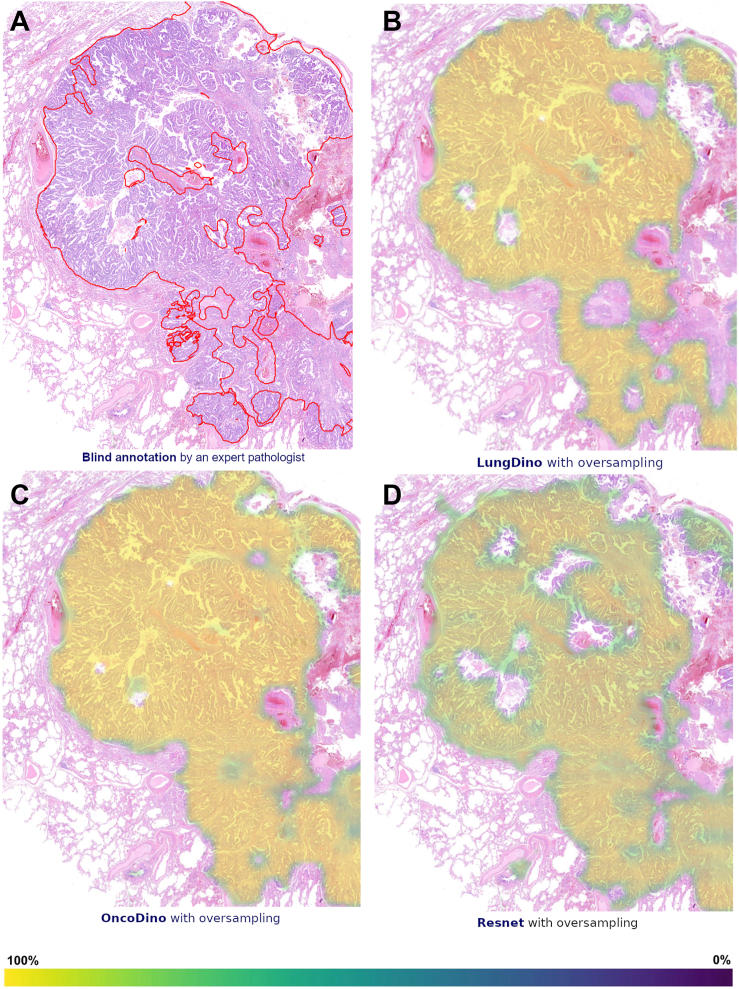
Tumor localization heatmaps generated by deep learning models in resection samples in comparison to expert pathologist annotation. Heatmaps indicate tissue areas most relevant for the final classification, highlighting probable tumor areas. The color gradient ranges from dark blue (0%) to bright yellow (100%), which corresponds to the attention or relevance score assigned by the model to each region. (*A*) Manual tumor annotation performed by a board-certified pathologist blinded to models’ outputs. (*B*) LungDino with oversampling. (*C*) OncoDino with oversampling. (*D*) The ResNet-based model.

The models ResNet, LungDino with oversampling, and OncoDino with oversampling were qualitatively evaluated for their ability to generate heatmaps on WSIs, highlighting tissue regions most relevant to their classifications. The results demonstrate the capability of the models to identify probable tumor areas. The probability of output is objectively classified as 0% to 100% and visually demonstrated in the heatmap by a range of colors from blue to yellow, as found in Figure 5 (biopsy samples) and Figure 6 (resection samples).

Blind Annotation by Expert Pathologist: The reference tumor region areas, manually annotated by an expert pathologist in a blind review, served as the ground truth for evaluating the accuracy and reliability of the model-generated heatmaps (Figs. 5*A* and 6*A*).

LungDino with Oversampling: The LungDino model with oversampling produced heatmaps that closely aligned with the expert pathologist’s annotations, demonstrating higher apparent specificity, although with slightly lower sensitivity in comparison to OncoDino (Figs. 5*B* and 6*B*).

OncoDino with Oversampling: OncoDino with oversampling also demonstrated strong performance, effectively localizing tumor regions. Compared with LungDino, its heatmaps appeared slightly broader, occasionally highlighting surrounding areas that were not identified as tumor by the pathologist, suggesting higher sensitivity but slightly lower specificity, particularly in the biopsy sample (Figs. 5*C* and 6*C*).

ResNet: The heatmap generated by the ResNet model revealed a reasonable, but somewhat limited correspondence with the pathologist’s annotations in the resection image, with lower apparent sensitivity and specificity, including lower probability, if compared with the other models (Fig. 6*D*). Tumor regions were inconsistently highlighted, and benign areas were more often misclassified. The model, although trained for this purpose, did not generate a heatmap in the biopsy sample (Fig. 5*D*).

## Discussion

To the authors’ knowledge, this study is the first to demonstrate the effectiveness of AI models trained on both global data sets and Latin American pathology samples for reliable subtype classification of HE-stained WSIs. Although the ResNet model achieved moderate performance, particularly struggling with squamous cell carcinoma, DinoV2-based feature extractors significantly outperformed it in both classification and tumor area identification tasks. Among these, LungDino achieved slightly higher overall accuracy for classification and heatmap generation. However, OncoDino demonstrated comparable performance and excelled in underrepresented categories such as small cell carcinoma. The oversampling strategy enhanced the performance of both models but introduced minor tradeoffs for other subtypes in the OncoDino model. Notably, both DinoV2 models achieved excellent performance in subtyping cases classified as poorly differentiated or undifferentiated in HE pathology reports, in contrast to the moderate performance observed with ResNet. These findings suggest that task-specific feature extraction can enhance accuracy, although general pathology feature extractors, such as OncoDino, offer broader applicability and generalizability across diverse data sets and tasks. Both approaches may represent valuable strategies for AI-assisted diagnosis in clinical practice, particularly for challenging cases.

Several studies have explored the use of deep learning for lung cancer pathology, focusing on tasks such as tumor area identification or subtype classification.^14^^,^^19^, ^20^, ^21^, ^22^, ^23^, ^24^, ^25^, ^26^, ^27^, ^28^ Most previous research has relied on convolutional neural networks (CNNs) such as AlexNet,^29^ GoogLeNet,^30^ ResNet,^14^ and VGGNet,^31^ typically benchmarked on the ImageNet data set.^22^ Although effective for some applications, CNNs are limited in their ability to capture long-term dependencies, which are critical for analyzing complex histologic images.^32^

The introduction of vision transformers for medical imaging tasks has demonstrated their potential in addressing these limitations. Unlike CNNs, transformers use self-attention mechanisms, allowing them to learn dependencies across entire feature sets.^32^ Vision transformers have been applied to various lung cancer imaging tasks, including classification, tumor segmentation, nodule detection, and survival prediction.^33^, ^34^, ^35^, ^36^, ^37^, ^38^, ^39^, ^40^, ^41^, ^42^ However, to the authors’ knowledge, no prior study has evaluated vision transformers for histologic subtype classification for lung cancer subtypes in the four main classes (adenocarcinoma, squamous cell carcinoma, small cell carcinoma, and benign tissue) or included Latin American samples in model development and testing, as what have been done in this study.

This study highlights the potential of DinoV2-based models to improve the classification of lung cancer subtypes and assist in tumor localization. By incorporating Latin American pathology samples into the training and testing of the models, the models address an important gap in AI-driven pathology research, increasing their relevance to underrepresented populations. The high classification accuracy and interpretability provided by heatmaps suggest that these models could complement existing diagnostic workflows by offering consistent and explainable insights to pathologists.

However, it is important to note that although this study includes a significant number of Latin American WSIs, the proportion of these samples in the overall training data set remains smaller compared with data sets such as TCGA and GTEx. This could limit the model’s ability to fully capture the representation of regional variability. In addition, the relatively small size of the test data set (79 WSIs) may restrict the generalizability of the study findings.

To address these limitations, the study’s team is currently developing a larger vision transformer Foundation Model using a more extensive data set of Latin American histologic samples. This model will be integrated with the classification pipeline and validated on a larger data set. Future studies should also explore integration of these models into clinical workflows to assess their impact on diagnostic efficiency, accuracy, and pathologist workload. Refinements in explainability tools, such as improving heatmap resolution and incorporating multimodal data, such as genomic or imaging data, could further enhance their utility in clinical practice.

## Conclusion

In this study, the effectiveness of two DinoV2-based feature extractors in accurately classifying lung cancer subtypes and localizing tumor regions using HE-stained WSIs was demonstrated. By incorporating Latin American pathology samples, a gap in AI research was addressed, enhancing the applicability and inclusivity of these models. The findings highlight the potential of self-supervised learning methods to improve diagnostic accuracy and reduce variability in histopathology, paving the way for more equitable and reliable AI-driven diagnostic tools. Future efforts will focus on expanding data sets and further validating these models to ensure their robustness and utility in clinical practice.

## CRediT Authorship Contribution Statement

**Viviane Teixeira Loiola de Alencar:** Conceptualization, Methodology, Validation, Data curation, Writing - original draft, Funding acquisition.

**Felipe Navarro Balbino Alves**: Conceptualization, Methodology, Validation, Software, Formal analysis, Writing - review & editing.

**Guilherme de Souza Velozo:** Investigation, Resources.

**Luiz Edmundo Lopes Mizutani:** Software, Formal analysis.

**Iusta Caminha:** Investigation, Resources. Gabriel Barbosa Silva: Investigation, Resources.

**Vladmir Cláudio Cordeiro de Lima:** Data analysis, Writing - review & editing, Supervision.

**Fábio Rocha Fernandes Távora**: Investigation, Resources, Data curation, Writing - review & editing, Supervision.

## Disclosure

Dr. Alencar, Mr. Alves and Dr. Távora are equity holders of Oncodata. Mr. Mizutani is supported by a research scholarship from 10.13039/501100001807FAPESP (São Paulo Research Foundation) for work conducted at Oncodata. The remaining authors declare no conflict of interest.
